# Impact of Concern About Falling on Physical and Social Activities of Older Adults With Hypertension

**DOI:** 10.1111/jgs.70030

**Published:** 2025-08-06

**Authors:** Dannah A. Shahin, Dan R. Berlowitz

**Affiliations:** ^1^ Zuckerberg College of Health Sciences; University of Massachusetts Lowell Lowell Massachusetts USA; ^2^ Boston University School of Medicine Boston Massachusetts USA

**Keywords:** concern about falling, falls, hypertension

## 1 Introduction

Nearly 50% of older community dwelling adults with hypertension are concerned about falling [1]. Studies have suggested that concern about falling may contribute to limitations in both social and physical activities [2, 3, 4, 5, 6, 7]. These studies, though, are mostly small [3, 5, 6], dated [2], from single locations [2, 5, 6], and in non‐US populations [3, 5, 6, 7]. The extent to which concern about falling is associated with reduced social and physical activities among people with hypertension has not been established. Due to the use of multiple antihypertensive medications, these patients may be particularly predisposed to orthostatic symptoms and increased fall risk. We now examine the impact of concern about falling using data from the Systolic Blood Pressure Intervention Trial (SPRINT).

## 2 Methods

Methods for SPRINT, a randomized clinical trial comparing intensive hypertension management to standard care, have been described elsewhere [8]. Sprint enrolled older people with hypertension from over 100 sites across the country. Concern about falling was captured using the Falls Efficacy Scale International (FES‐I), a seven‐item scale on which respondents indicate their level of concern when performing particular activities. Each item is scored on a 1 (not at all concerned) to 4 (very concerned) scale, and overall scores could range from 7 to 28. An overall score of 7 indicates no concern, 8–10 mild concern, and 11 or greater moderate to severe concern. Engagement in social activities was assessed with a single item asking how much of the time your health interfered with social activities, with responses ranging on a 5‐point scale from all of the time to none of the time. Daily time spent on moderate physical activities such as brisk walking or climbing stairs was quantified as less than 30 min, 30 min to 4 h, or greater than 4 h per day [9].

Associations between concern about falling (none, mild, moderate/severe) and both engagement in social activities and moderate physical activities were first examined with a chi‐square test. Regression models were then developed to examine the associations, adjusting for age, gender, race, and physical and mental health as measured by the VR‐12 health related quality of life scales [10].

## 3 Results

**FIGURE 1:**
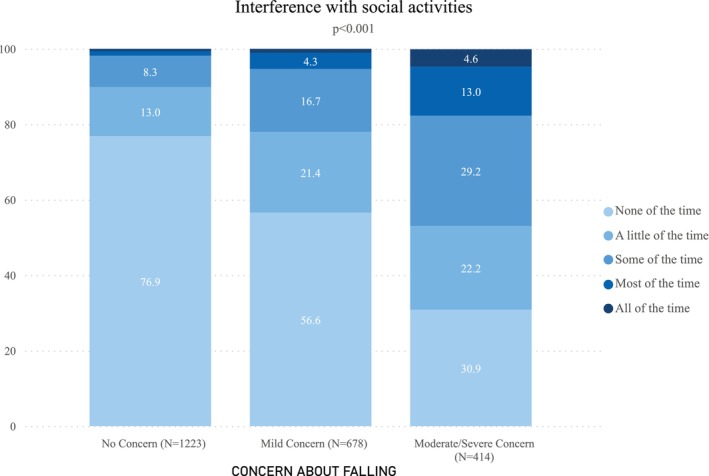
Interference with social activities due to health concerns as a function of concern about falling.

**FIGURE 2:**
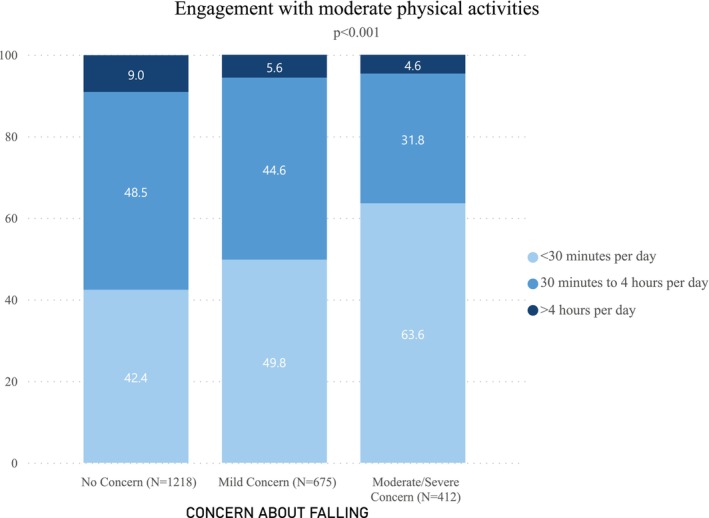
Engagement in moderate physical activities as a function of concern about falling.

The study sample consisted of 2315 individuals; due to missing responses, it was slightly smaller for physical activities. Some concern about falling was reported by 47.2%. Baseline characteristics have been described elsewhere [1]. The presence of concern about falling was associated with significantly (*p* < 0.001) less engagement in social and moderate physical activities (Figures 1 and 2). For example, among those reporting no concern about falling, 76.9% reported no limitations in social activities due to health concerns as compared to 30.9% with moderate/severe concern. Among those with no concern about falling, 42.4% reported engaging in less than 30 min per day of physical activity as compared to 63.6% of those with moderate/severe concern. In regression models, increasing concern about falling remained significantly (*p* < 0.001) associated with limitations in both social activities and engagement in moderate physical activities after adjusting for demographics, and physical and mental health status.

## 4 Discussion

These results suggest that concern about falling is associated with significantly reduced engagement in social and physical activities among older adults with hypertension. Participating in social activities is widely recognized as a key contributor to quality of life. Beyond the benefits of moderate physical activities on perceived health, in SPRINT it was also associated with a > 20% reduction in cardiovascular events and all‐cause mortality [9]. It could be that concern about falling is simply a marker for other causes of decreased social and physical activities such as older age or worse health. However, the association remained significant in regression models adjusting for these factors. It is important to emphasize that we have demonstrated an association rather than causality. A further limitation of this work is that there was no baseline data on fall history in SPRINT. Other studies have suggested, though, that falls may not be an important mediator of the association between concern about falling and quality of life [4]. Interventions to prevent falls and address concern about falling remain a priority in geriatrics research. Our results emphasize how these studies should also focus on secondary outcomes that are of importance to older adults such as engagement in social and physical activities.

## Author Contributions

Dan R. Berlowitz, affirm that I have listed all authors contributing to this work. Both authors contributed to study design, development of the analytic plan, interpretation of data, and drafting of the manuscript.

## Conflicts of Interest

The authors declare no conflicts of interest.
